# Recombinant Human Cytomegalovirus (HCMV) RL13 Binds Human Immunoglobulin G Fc

**DOI:** 10.1371/journal.pone.0050166

**Published:** 2012-11-30

**Authors:** Mirko Cortese, Stefano Calò, Romina D'Aurizio, Anders Lilja, Nicola Pacchiani, Marcello Merola

**Affiliations:** 1 Novartis Vaccines and Diagnostics, Siena, Italy; 2 Novartis Vaccines and Diagnostics, Cambridge, Massachusetts, United States of America; 3 Department of Structural and Functional Biology, University of Naples “Federico II”, Naples, Italy; University of Regensburg, Germany

## Abstract

The human cytomegalovirus (HCMV) protein RL13 has recently been described to be present in all primary isolates but rapidly mutated in culture adapted viruses. Although these data suggest a crucial role for this gene product in HCMV primary infection, no function has so far been assigned to this protein. Working with RL13 expressed in isolation in transfected human epithelial cells, we demonstrated that recombinant RL13 from the clinical HCMV isolates TR and Merlin have selective human immunoglobulin (Ig)-binding properties towards IgG1 and IgG2 subtypes. An additional Fc binding protein, RL12, was also identified as an IgG1 and IgG2 binding protein but not further characterized. The glycoprotein RL13 trafficked to the plasma membrane where it bound and internalized exogenous IgG or its constant fragment (Fcγ). Analysis of RL13 ectodomain mutants suggested that the RL13 Ig-like domain is responsible for the Fc binding activity. Ligand-dependent internalization relied on a YxxL endocytic motif located in the C-terminal tail of RL13. Additionally, we showed that the tyrosine residue could be replaced by phenylalanine but not by alanine, indicating that the internalization signal was independent from phosphorylation events. In sum, RL13 binds human IgG and may contribute to HCMV immune evasion in the infected host, but this function does not readily explain the instability of the RL13 gene during viral propagation in cultured cells.

## Introduction

Human cytomegalovirus (HCMV) infection is common and, although typically subclinical in the healthy population, it can cause severe disease in congenitally infected infants and in individuals with suppressed immunity [1]. In immunocompetent individuals, infection is controlled by both cellular and humoral immune responses, defenses that are weakened in immunocompromised patients leading to infection of several tissues and a vast range of cell types [2].

Characterization and use of clinical and low passage isolates of cultured HCMV strains have led to a reconsideration of the viral coding potential and suggested the presence of new unidentified functions (reviewed in [3]). Comparative sequencing analysis of unpassaged clinical isolates versus cell culture adapted viruses allowed a more refined identification of the genetic changes corresponding to functions that are lost in *in vitro* cultures. This is the case of RL13 and UL128 gene products, both possessing a suppressive phenotype on tissue culture adapted viruses [4], [5]. For RL13 in particular, Stanton *et al.* recently reported rapidly emerging genetic mutations following a few passages of BAC-derived Merlin strain virus in cultures of human cells of different origins [6]. These authors, using an elegant BAC system of conditional gene repression during virus propagation, were able to show that virus with reconstructed wild type RL13 repressed cell culture growth while the emerging deletion mutants allowed the virus to adapt to cell culture growth [6]. Providing that a functional RL13 gene appears to be carried by all sequenced clinical isolates, the authors hypothesized that this protein is critical for productive HCMV replication *in vivo*, perhaps increasing the repertoire of HCMV cell tropism [6].

HCMV has evolved a number of different ways to evade the immune system, often employing seemingly redundant factors and mechanisms to maintain the lifelong infection of the host [7]. Limitation of the host antibodies and complement activities through the expression of viral proteins able to bind the constant region of immunoglobulin G (Fcγ) is a mechanism common to several herpesviruses [8], [9]. These viral proteins interfere with the host receptors for the Fc portion (FcγRs) of immunoglobulin G (IgG), expressed on the surface of all cell types in the innate and adaptive parts of the immune system [10].

FcγRs are a complex family of proteins with several distinct classes and subclasses that function at the interface of the adaptive and innate immune systems [10]. They sense immune complexes in the extracellular environment and regulate signaling cascades in effector cells, which may contribute significantly to balancing pro- and anti-inflammatory responses to infection. HCMV expresses two proteins of 34 and 68 kDa that bind the Fc region of IgGs [11]–[13]. gp34 is encoded in clinical isolates by RL11 and in laboratory isolates by the duplicate genes TRL11 and IRL11 [12], [14]. gp68 is translated from the spliced mRNA encoded by UL119 and UL118 [11] and has been detected in preparations of purified virions [15]. Both gp34 and gp68 are glycosylated type I membrane proteins predicted to form immunoglobulin supergene family (IgSF)-like domains [11]. They bind all classes of human IgG with approximately equal affinity, whereas gp34 also binds rabbit IgG and, to a lesser extent, rat IgG1.

In this report we identified two additional Fcγ binding activities encoded by the HCMV genome, RL12 and RL13, and characterized this property of recombinant RL13 from the TR and Merlin strains of HCMV. We demonstrated that RL13 transfected into cultured human epithelial and fibroblast cells binds and internalizes the Fc portion of human IgGs.

## Results

### Fcγ binding ability of selected members of the RL11 family

**Figure 1:**
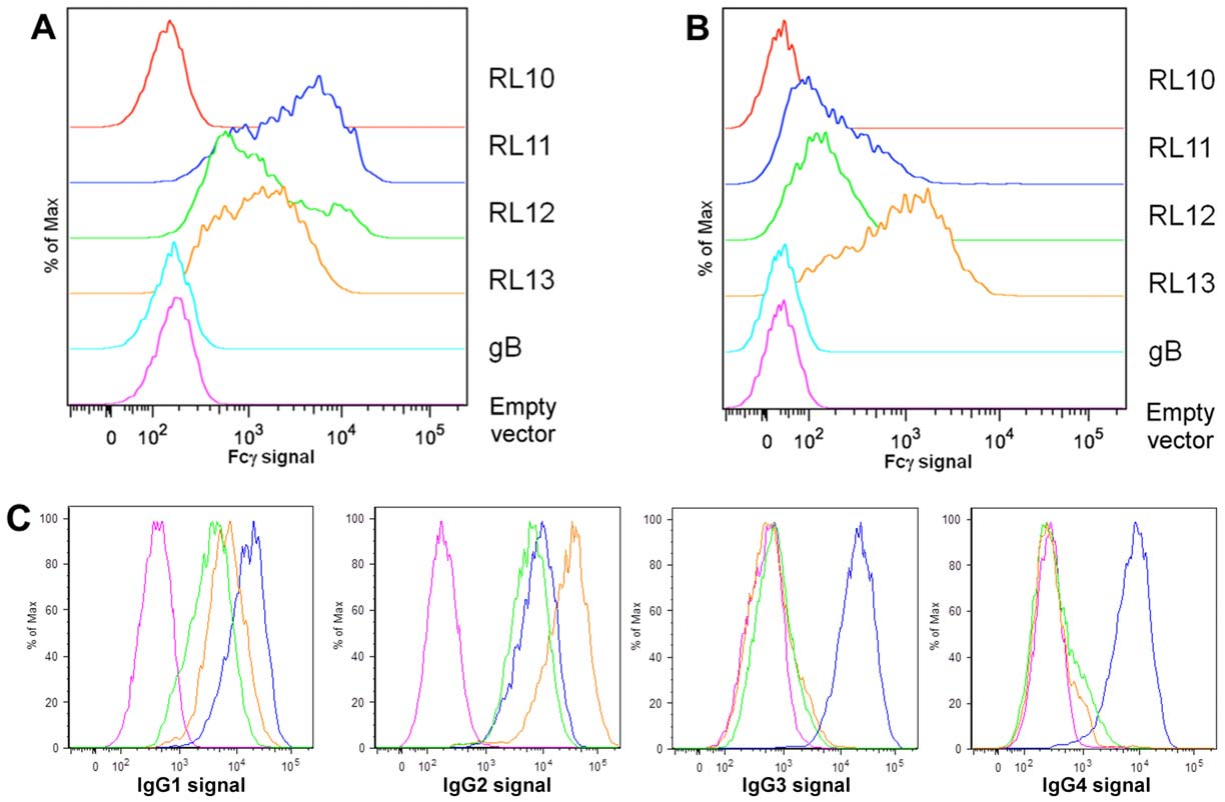
FACS analysis of Fcγ and IgG binding. **A**: Binding of Fcγ to RL13 in permeabilized cells. HEK293T cells transfected with empty vector or expression vectors for myc-tagged gB, RL10, RL11, RL12 or RL13 were fixed, permeabilized and stained using FITC-conjugated anti-myc and 25 µg/ml DyLight 649-conjugated human IgG Fc fragment (Fcγ). FITC positive cells were compared to mock transfected cells for their ability to bind Fcγ. **B**: Binding of Fcγ to surface-exposed RL13. HEK293T cells transfected with empty vector or expression vectors for myc-tagged gB, RL10, RL11, RL12 or RL13 were first stained at 4°C with DyLight 649-conjugated human IgG Fc fragment (25 µg/ml). Excess of probe was removed by washing in PBS and then cells were fixed and stained with FITC-conjugated anti-myc. FITC positive cells were compared to mock transfected cells for their ability to bind Fcγ. **C**: Binding specificity of RL10-RL13 towards different human immunoglobulin subclasses. HEK293T cells were transiently transfected with myc-tagged RL11 (blue), RL12 (green), RL13 (brown) or with empty vector (magenta). Cells were fixed, permeabilized and stained with FITC-conjugated anti-myc together with 10 µg/ml of human Ig of the different subclasses. Alexa fluor 647-conjugated goat anti-human was used as secondary antibody. FITC positive cells were compared to mock transfected cells for their ability to bind Fcγ.

RL11 was the first identified Fcγ binding protein of HCMV [12] and is hypothesized to function through its Ig-like domain [11]. Using RL11 as positive control, we sought to test if RL10, RL12 and RL13 were also able to bind human IgGs. We included gB (UL55) as a negative control. HEK293T cells were transfected with expression plasmids for myc-His tagged gB, RL10, RL11, RL12, RL13. 48 h after transfection, flow cytometry analysis was performed with DyLight 649-conjugated human Fcγ and FITC-conjugated anti-myc. Permeabilized (Fig. 1A) and non-permeabilized (Fig. 1B) FITC-positive cells were tested for ability to bind human Fcγ. RL11, RL12 and RL13 bind the Fc portion of human IgG while RL10 and gB are comparable to negative control (Fig. 1A and 1B). It appears that a large fraction of the Fcγ-binding activity of RL13 (brown trace) is surface exposed, whereas a large fraction of RL11 and RL12 (blue and green trace, respectively) is not (Fig. 1A and 1B). Expression of the different proteins was equal as demonstrated by comparable percentage of FITC positive populations and their mean fluorescence intensity (data not shown). Taken together, these data suggested that RL12 and RL13 are two previously unidentified human Fcγ binding proteins encoded by HCMV.

RL11 has been shown to bind all different subclasses of human IgGs [11]. To assess if RL12 and RL13 differentially recognized human IgG subclasses, flow cytometry on permeabilized HEK293T cells expressing RL11, RL12, or RL13 was performed using individual human IgG subclasses as probes. Cells expressing RL11 bind all IgG subclasses whereas cells expressing RL13 appear to specifically bind IgG1 and IgG2 (Fig. 1C).

These data and similar analysis of RL12 binding to human Ig isotypes and binding of RL12 and RL13 to Igs from different species are summarized in Table 1. Although the finding that RL12 binds Fcγ was novel, we focused the rest of this report on RL13.

**Table 1:** Ig-binding specificity of Fc-binding proteins RL11, RL12 and RL13.

		Ig binding by		
Species	Ig subtype	RL11	RL12	RL13
Human	IgG1	++	+	+
Human	IgG2	++	++	+++
Human	IgG3	++	-	-
Human	IgG4	++	-	-
Human	IgM	-	-	-
Human	IgA	-	-	-
Human	IgE	-	-	-
Rabbit	IgG	+	++	++
Mouse	IgG	-	-	-
Rat	IgG	-	-	-
Goat	IgG	-	-	-

HEK293T cells were transfected with expression plasmids encoding a tagged version of RL11 RL12, and RL13 genes, stained with the indicated immunoglobulins, and analyzed by flow cytometry. +/++/+++ indicates efficiency of IgG-binding observed as mean fluorescent intensity; – indicates no binding.

### Amino acid residues 109 to 218 of TR strain RL13 are necessary for Fcγ binding

**Figure 2:**
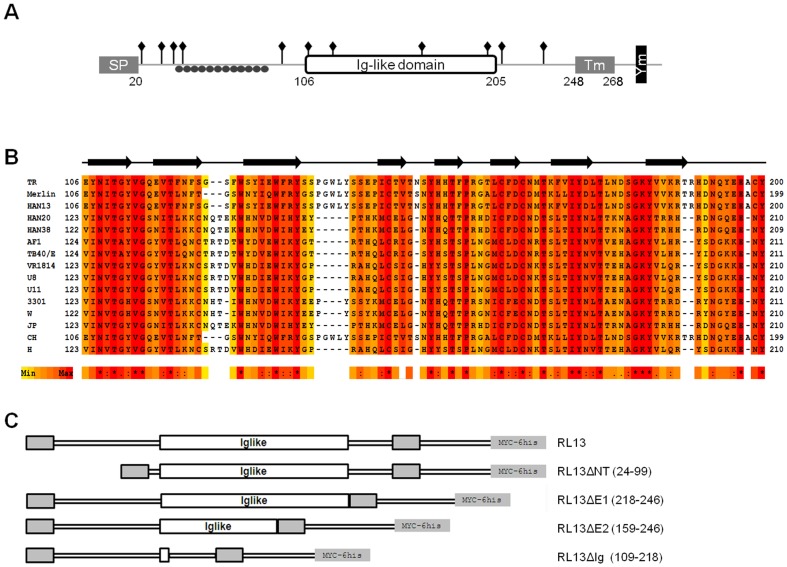
Predicted structure of the RL13 protein and schematic representation of TR RL13 ectodomain mutants. **A**: Schematic representation of the HCMV TR RL13 full length protein. The signal peptide at the N-terminus (SP), the Ig-like domain, the transmembrane domain (Tm), the tyrosine-based motif YxxL (Ym) sorting signal, 11 potential N-linked glycosylation sites (diamonds), and the O-linked glycosylation region (closed circles) are indicated. **B**: Multiple sequence alignment of the predicted RL13 Ig-like domain from the indicated strains of HCMV. The residues are colored according to the conservation level (red for higher conservation). Asterisks (*) below the alignments represent conserved amino acid in all sequences; colons (:) represent residues with similar physicochemical properties; dots (.) semi-conserved residues. The black arrows represent the positions of predicted β strands along the sequence. **C**: Graphic representation of TR RL13 and the ectodomain mutants used in this study. The amino-terminal signal peptide, the carboxy-terminal transmembrane (gray boxes) and the Ig-like domain (white box) are indicated.

The RL13 protein possesses strong topology similarities with the previously characterized HCMV Fcγ binding proteins coded by the RL11 and UL119 genes (gp34 and gp68 respectively) [11]. HCMV RL13 is a member of the RL11 multigene family [16] encoding a characteristic domain, known as RL11D or CR1 [17]. RL13 from HCMV strain TR has the typical features of a type I membrane glycoprotein (Fig. 2A): a 20 amino acids (aa) long signal peptide, a transmembrane domain in the 248–268 aa region, and a predicted cytoplasmic tail of 26 aa in accordance with previous studies [6]. Further analysis suggests the likely presence of 11 N-linked glycosylation sites (pos. 21, 32, 37, 41, 90, 108, 120, 167, 201, 208, 215 aa) and 22 O-linked glycosylation sites along the sequence, all restricted to the N-terminal region between residues 40 and 89. gp34 and gp68 contain a DxxxLL dileucine consensus motif for internalization and a potential I/V/L/SxYxxL intracytoplasmic immunoreceptor tyrosine based-like inhibition motif, respectively [11], [12]. Similarly, we noted the presence of a tyrosine-based motif (YxxL) for intracellular targeting of transmembrane proteins [18] in the C-terminal cytoplasmic domain of the RL13 sequence (Fig. 2A). A search for conserved patterns in the TR RL13 ectodomain revealed an immunoglobulin (Ig)-like domain (Fig. 2A) identified as SM00409 (IG) in the SMART family classification (106–205 aa, with an HMMSmart E-value of 6.16e-04) [19]. A multiple sequence alignment generated from 15 different HCMV strains reported a very high conservation along the entire domain except 3 short regions predicted to form loops (Fig. 2B). All 15 sequence variants contain an immunoglobulin domain identified as SMART SM00409 or Interpro IPR013783 (E-values ranging from 9.9e-07 to 3.4e-04 and from 4.70E-07 to 6.10E-04, respectively). The secondary structure predictions suggested a propensity of the identified TR RL13 Ig-like domain to form a series of 8 beta-strands (Fig. 2B).

To investigate the involvement of the Ig-like domain in the Fcγ binding ability, we produced 4 different mutants of the RL13 ectodomain (Fig. 2C): RL13ΔNT, lacking 76 residues (aa 24–99) from the N-terminal region predicted to contain the O-linked glycosylation sites; RL13ΔE1, lacking 29 (aa 218–246) residues between the Ig-like domain and the transmembrane region; RL13ΔE2 (aa 161–246), with the same deletion as above plus 57 residues of the C-terminus of the Ig-like domain; and RL13ΔIg, lacking the entire Ig-like domain (aa 109–218). The mutant proteins were transiently expressed fused to myc and His tags in HEK293T cells with comparable expression levels as judged by flow cytometry analysis using anti-myc antibody (data not shown).

**Figure 3:**
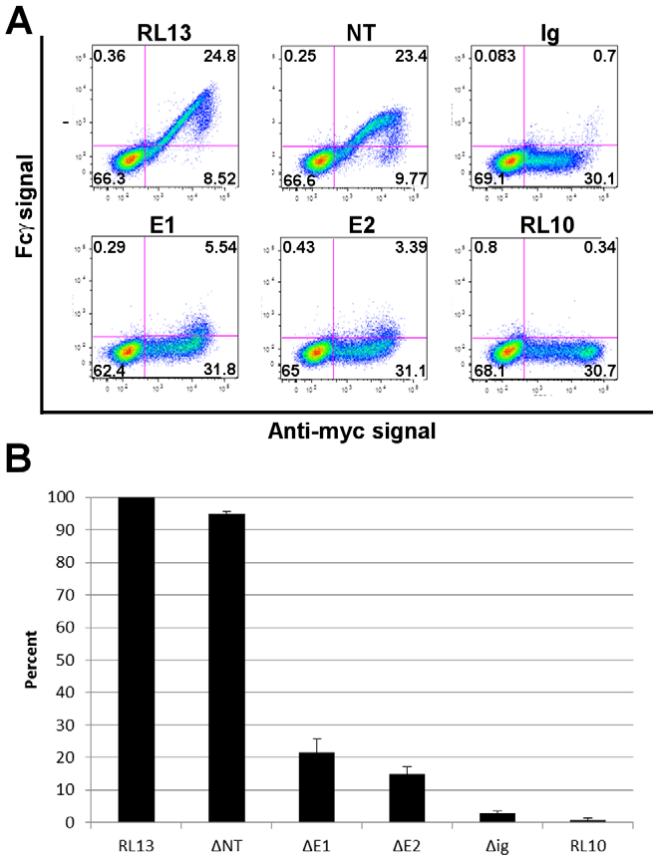
Fcγ-binding by RL13 ectodomain mutants. **A**: Cytofluorometric analysis of Fcγ binding by RL13 ectodomain mutants. HEK293T cells transfected with the indicated constructs were permeabilized and stained with DyLight 649-conjugated human IgG Fc fragment (Fcγ, 12.5 µg/ml) and Alexa Fluor 488-conjugated mouse anti-myc. Signals from 10,000 myc-positive cells are shown in each graph. The percentage of cells in each quadrant is indicated. **B**: Quantitative analysis of the FACS data. Each histogram shows the percentage of Fcγ positive cells relative to the RL13 wild type positive population (RL13). Values and error bars represent the mean and range of three independent experiments.

Fcγ binding ability was assayed by flow cytometry of myc-expressing permeabilized cells after staining with different concentrations of DyLight 649-conjugated Fcγ (Fig. 3A, only 12.5 µg/ml concentration is shown). Quantification of the results is reported as percentage of Fcγ stained cells in each sample compared to wild type RL13 (Fig. 3B). Values from RL13ΔNT were comparable to the wild type RL13, while a substantial reduction was observed in the RL13ΔE1 and RL13ΔE2 samples. Deletion of the Ig-like domain carried by the RL13ΔIg (RL13ΔE1 and RL13ΔE2) mutants completely abolished specific Fcγ binding since the number of positive cells was comparable to negative controls, cells expressing RL10 or transfected with empty vector (Fig. 3A–B).

These data indicated that the Fcγ binding activity of RL13 maps to amino acids 109–218. Removal of the region located between the Ig-like domain and the transmembrane domain severely impaired Fcγ binding but did not completely abrogate it. This observation suggests that this region could be important for the stability of the binding and/or the correct protein fold.

### Subcellular localization of RL13 in transfected cells

Stanton *et al.* recently reported that adenovirus-expressed RL13 transits through the Golgi and traffics mainly to Rab5-positive cytoplasmic vesicles [6]. We sought to verify the intracellular localization of RL13 expressed transiently in human cells. Confocal microscopy images were collected from epithelial (ARPE-19) and fibroblast (MRC-5) cells transfected with two different constructs, the previously described myc-His tagged construct and a second construct coding for RL13 with a C-terminal YFP-tag. Since sub-cellular localization of RL13 with different tags was identical in both cell types (data not shown), only representative data obtained in ARPE-19 human epithelial cells with RL13-YFP are shown.

**Figure 4:**
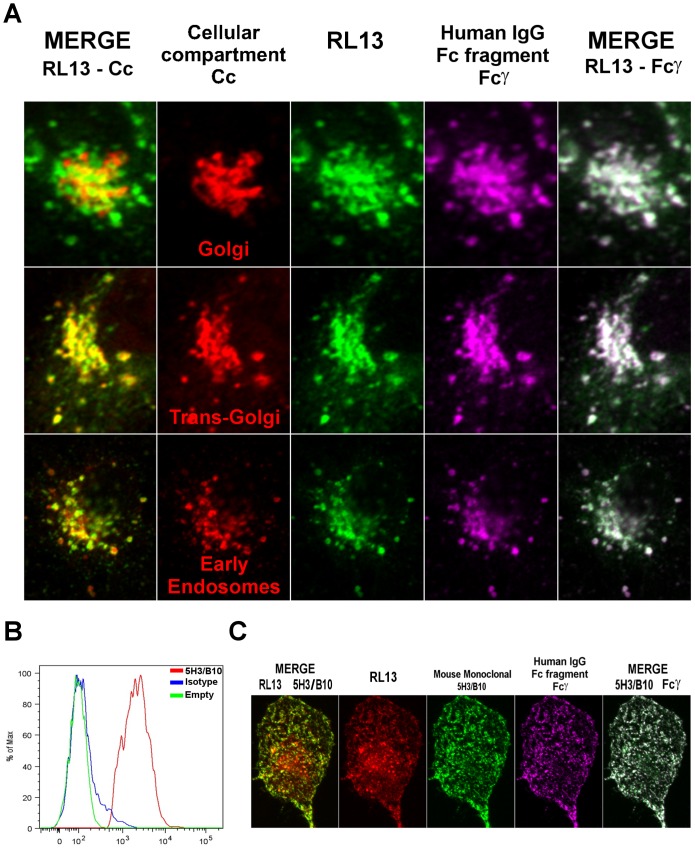
Localization of RL13 in transfected cells. **A**: Co-localization of RL13 with organelle markers and Fcγ in ARPE-19 cells. ARPE-19 epithelial cells were transfected with RL13-YFP fusion protein (green color, central column) and treated for confocal analysis 48 h later. Cells were fixed, permeabilized and stained with antibodies against either GM130, TGN46 or EEA1 intracellular markers (red color, second column) and with 20 µg/ml of DyLight 649-conjugated human IgG Fc fragment (magenta color, fourth column). The merged panels on the far left show co-localization between RL13 and, from top to bottom, markers of Golgi (GM130), trans-Golgi (TGN46) and early endosomes (EEA1) respectively (co-localization in yellow). The merged panels on the far right show co-localization of Fcγ with RL13 (co-localization in white). **B**: Flow cytometry analysis of RL13 surface-expression. HEK293T cells were transiently transfected with vector coding for YFP (green line) or RL13-YFP fusion protein (red and blue lines). Cells were allowed to recover for 48 h and then surface-exposed RL13 was stained either with 5 µg/ml of mouse monoclonal antibody directed against RL13 ectodomain (clone 5H3/B10, red and green lines) or isotype control at the same concentration (isotype, blue line) on ice. Alexa fluor 647 conjugated goat anti-mouse was used as secondary antibody. YFP positive cells were compared to empty vector transfected cells for their ability to bind 5H3/B10 antibody. **C**: Immunofluorescence analysis of RL13 surface expression on ARPE-19 cells. ARPE-19 cells expressing RL13-YFP fusion protein (red color) were stained on ice with 12.5 µg/ml of 5H3/B10 monoclonal antibody (green color) and 20 µg/ml of DyLight 649-conjugated human IgG Fc fragment (magenta color) without permeabilization. Merge panels show co-localization between 5H3/B10 and either RL13-YFP or Fcγ signals (yellow color in the far left panel and white color in the far right panel, respectively).

48 h after transfection, cells were fixed, permeabilized and stained with different markers of cellular compartments and with fluorophore-conjugated Fcγ. The confocal microscopy analysis of these samples is shown in Fig. 4A. RL13 co-localized to a limited extent with Golgi markers and EEA1-positive endosomes and more extensively with markers of the trans-Golgi network (TGN) (Fig. 4A, left panels). Fcγ co-localized extensively with RL13 (Fig. 4A, far right panels), although a population of RL13 that does not bind to the Fc of human IgGs was consistently observed (Fig. 4A, green color in far right panels). Although it is tempting to speculate that this pool represents an immature ER population, also suggested by the shape of the RL13 distribution, we have been unable to find any co-localization of RL13 with the ER marker PDI (not shown).

The presence of RL13 on the surface of both HEK293T and ARPE-19 cells was further verified by the use of a mouse monoclonal antibody (mAb 5H3/B10) directed against the ectodomain portion of RL13. Analysis by flow cytometry was performed on transiently transfected non-permeabilized HEK293T cells (Fig. 4B). A clear increase in fluorescence was obtained on RL13 expressing cells stained with mAb 5H3/B10 compared to mock transfected cells or isotype control. Confocal microscopy analysis of non-permeabilized RL13 expressing ARPE-19 cells stained with both Fcγ and 5H3/B10 revealed a strong accumulation of RL13 on plasma membrane clusters (Fig. 4C) that extensively overlapped with Fcγ signals (Fig. 4C, white color in right merge panel).

These observations are consistent with RL13 trafficking through the secretory pathway and recycling from the plasma membrane, although other possible explanations for this pattern have not been excluded.

### Internalization of RL13-Fcγ complex

**Figure 5:**
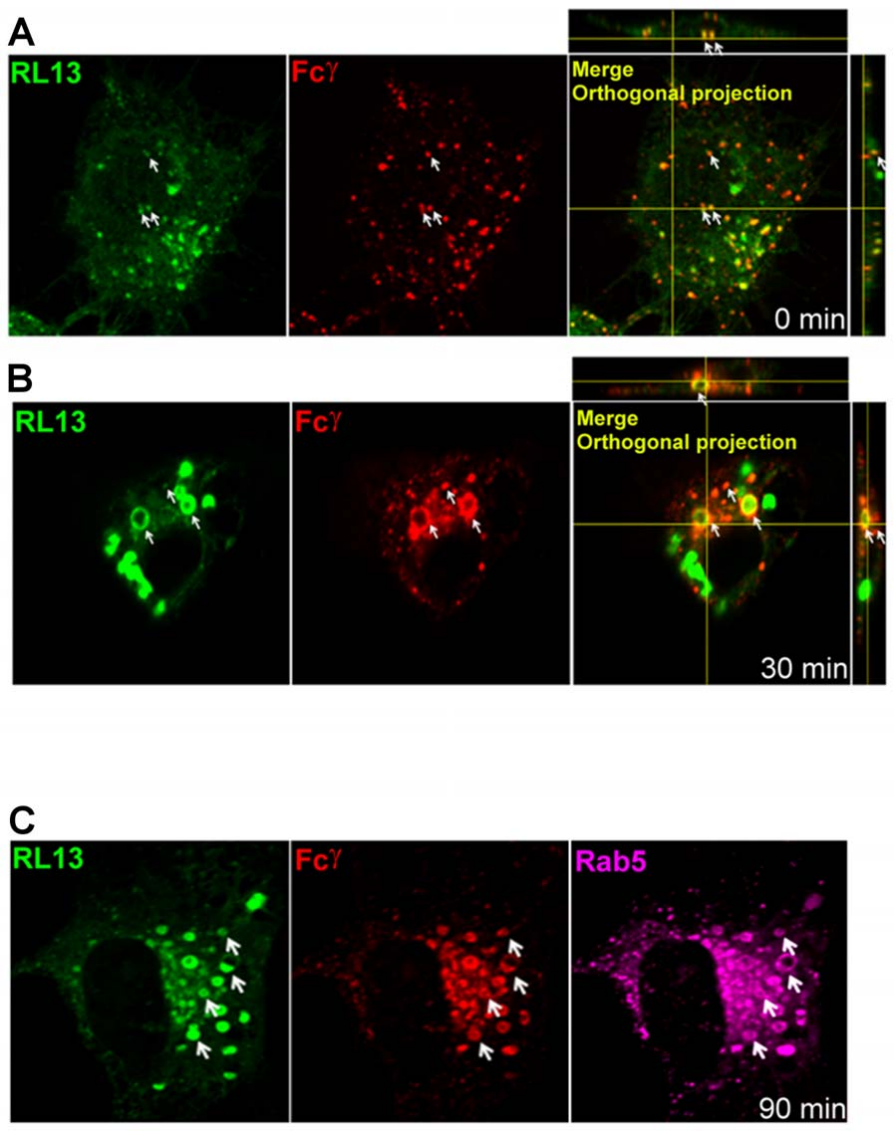
RL13 binds and internalizes Fcγ. ARPE-19 epithelial cells were transfected with RL13-YFP (green) and allowed to recover for 48 h. Cells were placed on ice 5 min before adding DyLight 649-conjugated human IgG Fc fragment (red) and incubated on ice for 30 min. Then, cells were washed and **A**: immediately fixed, **B**: shifted to 37°C for 30 min, or **C**: shifted to 37°C for 90 min before proceeding to fixation and confocal analysis. Rab5 (purple) was revealed by specific antibodies after permeabilization of the samples. Examples of co-localization between signals are indicated by white arrows. Orthogonal projections of the optical sections acquired from Z-stack are shown. White arrows indicate examples of co-localized spots. Fluorescent Fcγ was absent on the membrane of ARPE-19 cells transfected with empty expression vector used as control and no staining was detected following 30 min switch at 37°C (data not shown).

The presence of an internalization motif in the C-terminal tail, together with the intracellular localization data in Fig. 4, suggested that RL13 could traffic to the cell surface where it may bind and internalize extracellular IgGs. To test this hypothesis, we performed a short time-course characterization of the internalization of YFP-tagged RL13 in transfected ARPE-19 cells. Cells were initially placed on ice to reduce lateral diffusion of membrane proteins and block potential internalization of the ligand. Fluorescently labelled Fcγ was added and allowed to bind for 30 min on ice. Following extensive washing of the Fcγ excess and fixation of the cells, fluorescence analysis at time 0 showed that RL13 partially co-localized with Fcγ on the surface of transfected cells (Fig. 5A). Restoring the internalization processes at 37°C induced the uptake of the RL13- Fcγ complex, the majority of which accumulated mostly in large ring-shaped structures after 30 min (Fig. 5B). These RL13-Fcγ structures persisted 90 min after the shift to 37°C and stained positive for the early endosome marker Rab5 (Fig. 5C).

These data demonstrate that RL13 binds to the Fc of human IgGs on the cell surface and that the internalized complex remains associated with large membranous vesicles containing markers of early endosomes.

### Mutation of RL13 sorting motif affects the internalization of Fcγ

**Figure 6:**
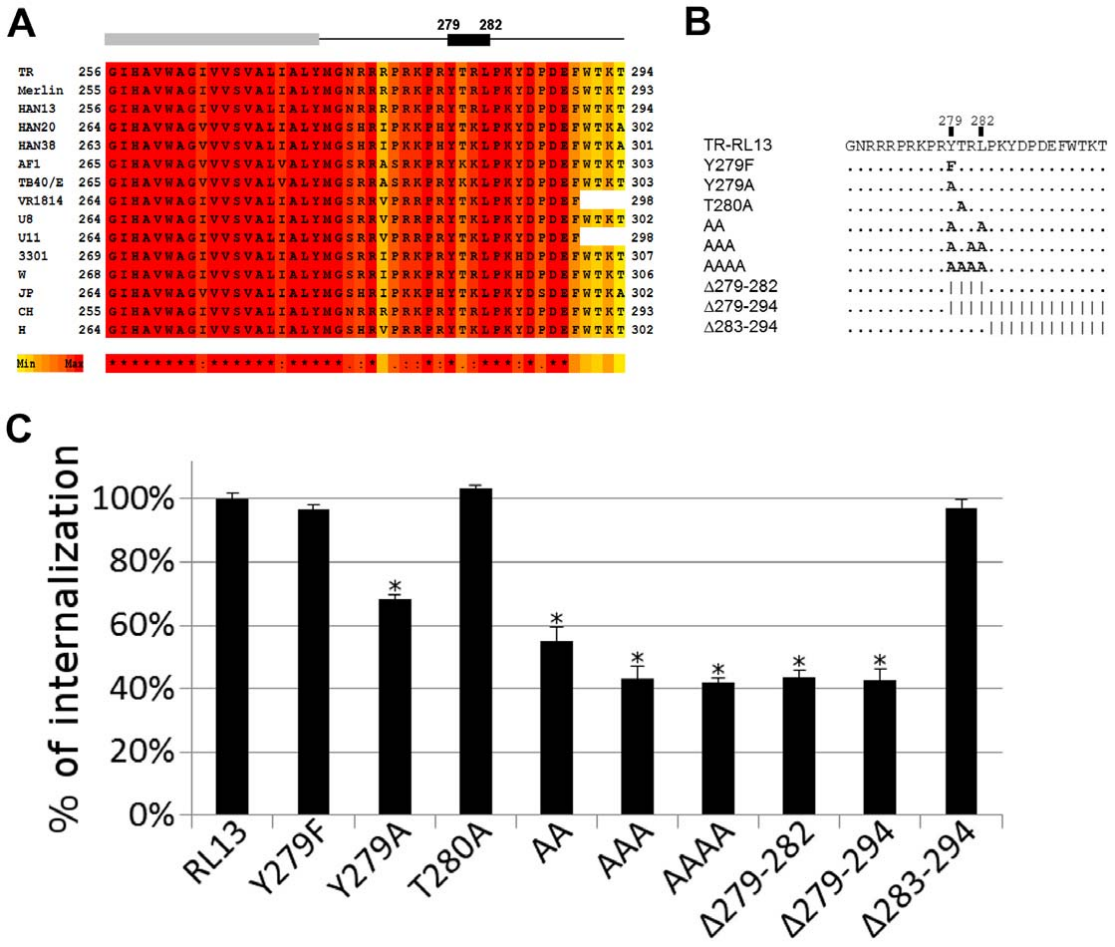
RL13 C-terminal YxxL motif is required for internalization. **A**: Local multiple sequence alignment of the RL13 C-terminus. The gray box indicates the transmembrane domain, the black box the YxxL internalization motif. Darker color indicates higher conservation. Asterisks (*) below the alignments represent conserved amino acid in all sequences; colons (:) represent residues with similar physicochemical properties; dots (.) semi-conserved residues. **B**: Graphical representation of RL13 mutations used in this study. RL13 TR cytoplasmic tail sequence is shown with mutated amino acids in bold. Amino acids unchanged from the wild-type sequence are represented by a dot. Deleted amino acids are represented by a |. **C**: Internalization efficiency of RL13 variants. HEK293T cells were transfected with the indicated plasmids coding for wild type and mutated RL13. 48 h post-transfection, cells were detached and incubated on ice for 60 min with human Fcγ fragment. Cells were then washed and incubated in medium at 37°C for 30 min to allow for endocytosis (T37). The control sample remained on ice (T0). Following incubation, cells were cooled quickly by rinsing twice with cold PBS; Fcγ that remained on the cell surface after endocytosis was stained with Alexa Fluor 647-conjugated goat anti-human IgG and analyzed by flow cytometry. The % of internalization was calculated from the mean fluorescence intensities of transfected cells with the following formula: (T0–T37)/T0×100%. Value retrieved from RL13 wild type was set to 100%. Values are the mean and range of three independent experiments. Significant differences, *P*<0,001 (two-tailed unpaired Student's t-test), compared to RL13 are indicated by *.

As shown by the *in silico* analysis, the carboxy-terminal domain of RL13 contains a tyrosine-based sorting signal (YxxL_282_) that could be responsible for the internalization of the RL13-Fcγ complex. A local multiple sequence alignment of the C-terminal region of RL13 from 15 HCMV strains showed that the YxxL motif was highly conserved (Fig. 6A, residues 279–282). To test the contribution of the cytoplasmic tail, and in particular of the tyrosine motif, to the internalization of the RL13-Fcγ complex, a panel of mutants were built (Fig. 6B). Plasmids encoding RL13 with three single amino acid substitution mutants, Y279F, Y279A, T280A, one double substitution mutant Y279A/L282A (indicated as AA), one triple substitution mutant Y279A/R281A/L282A (AAA), and one quadruple substitution mutant with all amino acids of the motif replaced by alanines (AAAA) were produced. Additionally, plasmids encoding a YxxL deletion mutant (Δ279–282), a deletion of the motif and the entire C-terminal (Δ279–294), and a deletion of 12 amino acids immediately C-terminal to the sorting motif (Δ283–294) were generated. 48 h after transfection, internalization of the mutated/deleted proteins was assessed by flow cytometry on HEK293T cells incubated with Fcγ at 4°C for 30 min and, after removal of the ligand by extensive washing, shifted to 37°C. The percent of internalized Fcγ was calculated from the mean fluorescence intensity of the samples incubated at 37°C for 30 minutes and the samples submitted to the same treatment but kept on ice. As shown in Fig. 6C, the YxxL motif is critical for protein internalization since removing the region downstream did not alter the RL13 Fcγ internalization ability while deletion of the motif, both individually or with the C-terminus sequence, strongly reduced the internalization ability of RL13 (Fig. 6C). Substitution of the tyrosine 279 with phenylalanine, an alternative aromatic side chain, did not reduce the ability of RL13 to internalize. In contrast, uptake was reduced by approximately 1/3 when an alanine was placed in this position. Substitution of the adjacent threonine 280 with alanine did not show a difference in internalization compared to the wild type. However, leucine 282 appeared to play a role as shown by the further internalization reduction of the Y279/L282 double mutant (Fig. 6C). Substitution of multiple residues with alanine reduced internalization to the same extent as complete deletion of the YxxL motif (Fig. 6C).

Taken together these results are consistent with the YxxL_282_ motif being necessary for Fcγ internalization by RL13 and suggest that an aromatic residue at position 279 is required.

### Fcγ binding ability is conserved in the HCMV Merlin strain

**Figure 7:**
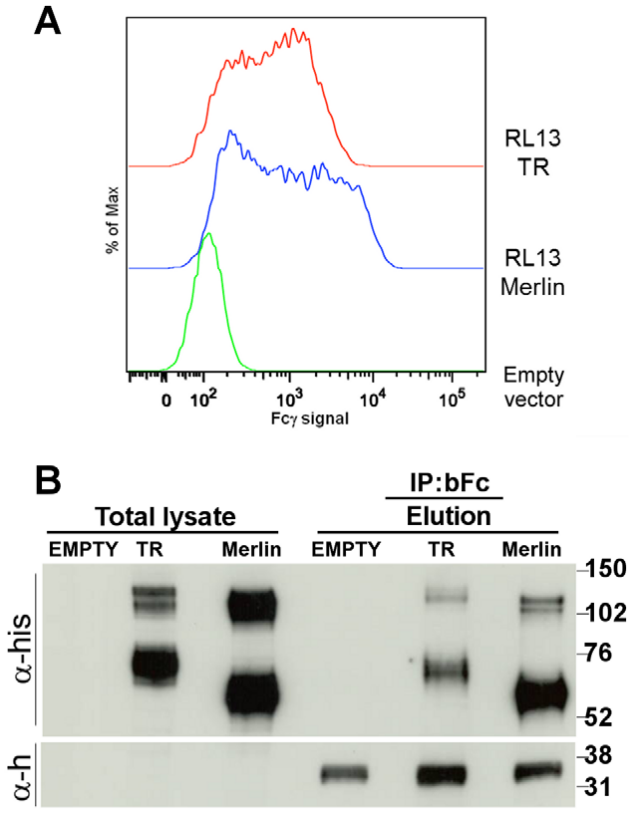
Fcγ binding is a conserved function for recombinant RL13 from Merlin and TR clinical strains. **A**: HEK293T cells were transfected with plasmids coding for myc-His tagged RL13 from TR (red), Merlin (blue) strains and empty vector (green). 48 h post-transfection cells were collected, permeabilized and stained with FITC-conjugated mouse anti-myc and 25 µg/ml of DyLight 649-conjugated human IgG Fc fragment. 10,000 FITC-positive cells were compared to empty vector transfected cells. **B**: HEK293T cells were transfected with empty vector or plasmids coding for myc-His tagged RL13 from TR and Merlin strains. 48 h post-transfection cells were collected, lysed and subjected to immunoprecipitation with biotinylated Fcγ fragment. Total lysates and elution fractions were subjected to western blot analysis using HRP-conjugated anti-his tag (α-his) and anti-human IgG (α-h) antibodies. TR: sample expressing myc-His tagged RL13 from TR strain; Merlin: sample expressing myc-His tagged RL13 from Merlin strain; Empty: empty vector transfected cells.

Due to the high variability of the RL13 genes among different HCMV strains [20] we also tested the Merlin RL13 protein (87% aa identity with its TR counterpart) for its Fcγ binding ability. *In vitro* synthesized Merlin RL13 gene was cloned in pcDNA3.1(-)/myc-His expression vector and used to transfect HEK293T cells. Intracellular staining was performed as previously described and cells were analyzed through flow cytometry. Both the expression levels (data not shown) and the Fcγ binding ability of cells expressing RL13 from Merlin and TR strains were comparable (Fig. 7A). To further confirm the specificity of the binding, both proteins were immunoprecipitated from transfected cell lysates with biotinylated Fcγ. Complexes were captured with magnetic streptavidin beads and eluted material was separated by SDS-PAGE and analyzed by immunoblot using anti-tag and anti-human IgG antibodies (Fig. 7B). Both Merlin and TR RL13 proteins were successfully immunoprecipitated from cell lysates. The observed molecular weights of the two proteins in denaturing and reducing conditions appeared to be slightly different: TR RL13 was composed of three major isoforms of around 123 kDa, 111 kDa and 76 to 66 kDa, while Merlin RL13 gave three bands of 116 kDa, 108 kDa and 66 to 52 kDa. These differences in the molecular weight could reflect the different glycosylation profiles, with 7 and 11 predicted N-linked glycosylations for RL13 from Merlin and TR strains, respectively.

These experiments support the conclusion that the Fcγ-binding ability of RL13 is conserved in at least two different clinical HCMV isolates.

## Discussion

The low fitness of wild-type RL13-carrying strains cultured *in vitro*, leading to the destruction of the ORF present in the clinical isolates within a few passages [5], [6], make functional studies of this protein difficult. To better understand its function, we used synthetic RL13 genes reproducing the sequences of the clinical isolates TR and Merlin. In the absence of an unpassaged reference sample of TR, we cannot say with certainty that the sequence and function of the TR RL13 we used is identical to the originally isolated, naturally occurring virus. However, we do not detect functional differences between RL13 from the TR and Merlin strains. Expression of this recombinant protein in human-derived epithelial cells allowed us to demonstrate that RL13: a) binds Fc from IgG of human (and rabbit) origin; b) preferentially binds to Fc from IgG1 and IgG2 subclasses; c) is at least transiently exposed on the cell surface and is subsequently internalized with (and likely without) bound Fcγ; d) internalization relies on a YxxL motif and e) its Ig-like extracellular domain is necessary for Fcγ binding. Similar analysis of RL13 in human fibroblasts produced identical results (data not shown). Furthermore, we revealed that RL12 also binds IgG1 and IgG2, although we did not study this interaction in detail. Our data does not offer an explanation for why RL13 is so rapidly mutated when clinical isolates of HCMV are grown in cultured cells [5].

Apart from HCMV, viral Fcγ binding proteins (vFcBPs) are present on herpes simplex virus type 1 (HSV-1), HSV-2, and varicella zoster virus and on the non-human herpesviruses pseudorabies virus and murine cytomegalovirus [21]–[25]. The most accepted model to explain how those activities promote immune evasion is the so called “antibody bipolar bridging” in which the neutralizing potential of the IgG Fab domain is counteracted by simultaneous binding of vFcBPs to the Fc domain of the same immunoglobulin molecule [26], [27]. The possibility of this mechanism has been supported at a molecular level by Sprague and coworkers [27], who crystallized a portion of HSV gE ectodomain and gE-gI complex bound to an human IgG Fc fragment. Alternatively, it has been hypothesized that HSV, as well as HCMV vFcBPs, function once expressed on infected cell surface by endocytosis and subsequent degradation of complexes including human IgG and viral antigens [11], [27]. Consistent with this hypothesis, we observed that RL13 is able to internalize human IgG Fc fragment from the surface of transfected cells trafficking at early times in Rab5 and EEA1 positive endosomes. The well-recognized YxxL motif is essential for RL13 endocytosis through the aromatic nature of the tyrosine and not its property of phosphate acceptor. As already described for the RID receptor of adenovirus [28], this type of sorting signal does not affect signal transduction pathways. On the other hand, the fact that RL13 is located on the envelope of the virus [6] suggests that antibody bipolar bridging could be an important function of RL13 that would help protect HCMV virions from complement- and antibody-dependent neutralization.

RL13 and RL12 discriminate among human IgG subtypes showing no association with IgG3 and IgG4. To our knowledge, this observation remains the only example of vFcBPs selecting not only among immunoglobulin isotypes but also IgG subtypes. IgG2 represents roughly 25% of human total plasma IgGs in adults and has been traditionally associated with protection from infection with encapsulated bacteria since their immune function is primarily directed against polysaccharide antigens [29]. Recent reports, however, have pointed out a significant association between IgG2 deficiency and severe or even fatal clinical outcome of the pandemic influenza A (H1N1), although IgG2 unbalance has been suggested to be a consequence of cytokine deregulation rather than a predisposing risk factor [30], [31]. Due to the low number of studies performed on human IgG2 function related to viral infection, the meaning of such observation remains elusive and needs further analysis. Compared to IgG1, subtype 2 immunoglobulins cannot mediate complement-dependent cytotoxicity or NK-mediated antibody-dependent cellular cytotoxicity (ADCC) but do engage FcγRIIa receptors and trigger ADCC mediated by cells of the myeloid lineage [32]. Again, the relative importance of IgG2 subtype in protecting from viral infection just begun to be addressed and it is difficult to evaluate the importance of a viral factor able to counteract such mechanism, if any, but it is noteworthy that HCMV has a latent reservoir in myeloid cells [33]. It is tempting to speculate that a viral IgG2-binding receptor could compete with cellular receptors for binding to cellular antibodies to viral proteins and prevent ADCC. It would be important to determine the affinity of RL13, as well as of the other HCMV Fcγ binding proteins, toward IgG2 to evaluate if this protein represents a function more dedicated to a particular IgG subtype.

Atalay and coworkers described the presence of immunoglobulin supergene family (IgSF)-like domains in the products of both RL11 and UL119 genes [11]. We have mapped the RL13 Fcγ binding activity to the Ig-like domain in the extracellular region, while the N-terminal proximal region composed of approximately 70 amino acids is not crucial for the binding. Indeed, Sprague *et al.* showed that removing the N-terminal part of the gp68 ectodomain did not impair the Fcγ binding [13]. These data are consistent with the presence of an immunoglobulin supergene family (IgSF)-like domains in all the described HCMV Fc binding proteins. Our findings do not rule out the possibility that RL13 could carry out other functions. The best characterized viral Fc binding protein, the gE product of HSV-1, is also involved in cell-to-cell spread or trafficking of viral proteins from neuron body cell into axon independently of its Fc binding capability [34], [35]. We are currently investigating other possible roles of RL13 during HCMV pathogenesis.

Our preliminary characterization of transiently transfected RL13 agrees only partially with what was reported by Stanton *et al.* [6]. They found significant differences in cellular localization and apparent SDS-PAGE MW between adenovirus-expressed RL13 and the protein expressed in the context of viral infection. In their report, adenovirus-expressed RL13 showed a lower MW (80 and 55 kDa) compared to the species found in infected cells (100 and 55 kDa) and the protein only co-localized with TGN in the context of infection [6]. The behavior of RL13 observed in this report is more similar to Stanton *et al.*'s data for infected cells, showing a MW above 100 kDa and a sub-cellular co-localization with early endosomes and the TGN. This last observation would be consistent with a default mechanism of internalization similar to what was described for other HCMV envelope proteins [36]. We currently have no explanation for the slight discrepancies between Stanton *et al.* and our report that may be due to the different expression system used or, at least for the confocal analysis, to the protein derived from a different strain. However, characterization of the expressed RL13 remains preliminary and further studies will elucidate this point.

To conclude, we have shown that RL13 encoded by HCMV TR binds the Fc portion of human IgG1 and IgG2 and propose that this protein is used by the virus to circumvent the humoral immune response in the host.

## Materials and Methods

### Sequence data, in silico analysis and predictions

Start to stop open reading frames (ORFs) in the complete genome sequence of the TR strain (Genbank: AC146906.1) were identified using the Getorf program from the EMBOSS suite 5.0.0 [37]. The sequence similarity searching algorithm FASTA 35.4.3 [38]. was exploited to compare the protein sequences of RL13 gene product from the Merlin strain (Genbank: YP_081461.1) to all the TR ORFs and select homologous ones with BLOSUM50 as substitution matrix and imposing an expectation value (E) upper limit of 1e-05. The putative TR RL13 sequence was searched for specific conserved signatures using InterProScan 4.8 [39] with databases v.34.0. Multiple sequence alignments were performed using the PSI-Coffee mode of T-Coffee v.9.01 [40], comparing the TR RL13 with an additional 14 different RL13 protein sequences (Genbank: YP_081461.1, AEI84615.1, ACS91939.1, AAR31271.1, AAR31220.1, ACT81850.1, ACT81685.1, ACS92104.1, ACZ79760.1, ACZ79925.1, ADI46773.1, ACZ80255.1, ABV71530.1, ACZ80090.1). Signal peptides and transmembrane regions were predicted by Phobius [41], while secondary structures were predicted using PSIPRED [42]. N-linked and O-linked glycosylation sites were predicted using NetNGlyc 1.0 (http://www.cbs.dtu.dk/services/NetNGlyc/) and NetOGlyc 3.1 [43] (http://www.cbs.dtu.dk/services/NetOGlyc/) respectively.

### Cells, plasmids and antibodies

ARPE-19, MRC-5 and HEK293T cell lines were brought from ATCC and cultured according to the supplier's instructions. DMEM high glucose and DMEM:F12 media (Gibco, Invitrogen) were supplemented with 10% fetal calf serum (FCS) and penicillin streptomycin glutamine (Gibco, Invitrogen). Lipofectamine 2000 (Invitrogen) was used to transfect HEK293T cells while Fugene 6 (Roche) and Nucleofector kit V (Amaxa) were used to transfect ARPE-19 cells as suggested by manufacturer. Human codon-optimized RL10, RL11, RL12, RL13 and gB (UL55) HCMV genes based on the TR strain sequence and RL13 from Merlin strain (NCBI Reference Sequence: YP_081461.1) were synthesized by Geneart and cloned in plasmid pcDNA3.1(-)/myc-His C (Invitrogen) in frame with C-terminal myc and six histidine tag sequences. Fluorescent fusion proteins were obtained by subcloning a gene of interest upstream of the EYFP sequence in pEYFP-N1 vector (Clontech). Ectodomain and C-terminal tail RL13 mutants were cloned using QuickChange™ Site-directed Mutagenesis kit and instructions therein (Stratagene). Primary antibodies used in this work were mouse anti-His (C-term) and mouse anti-PDI (Invitrogen), mouse anti-Rab5 mouse, anti-GM130, mouse anti-TGN46 and mouse anti-EEA1 (Abcam). Mouse monoclonal antibody against RL13, clone 5H3/B10, was developed by Areta International (Gerenzano, Italy). Mice immunization was performed with peptides encompassing the amino acid region 111–246 of TR strain RL13. Secondary antibodies were Alexa Fluor 488-, 568-, and 647-conjugated goat anti-mouse (Invitrogen) and HRP-conjugated secondary antibodies from Perkin Elmer. Chrompure DyLight 649-conjugated rat, rabbit, mouse, goat IgG and human Fc fragment were from Jackson Immunoresearch, human IgG subclasses from SIGMA, and human IgA, IgE, IgM from Calbiochem.

### Flow cytometry

For intracellular staining, HEK293T cells transfected with the plasmids of interest were trypsin detached 48 h post-transfection, incubated 30 min at RT with Live&Dead Agua (Invitrogen), diluted 1∶400 in PBS, fixed and permeabilized with Cytofix/Cytoperm kit (BD) and stained as instructed by the manufacturer. For cells expressing the myc-tagged proteins, mouse anti-myc-FITC antibody was used at 1∶500 dilution. Binding of the Fc portion of the human IgGs (Fcγ) was assessed using DyLight 649-conjugated human IgG Fc fragment or different human immunoglobulin isotypes and human IgG subclasses at different concentrations (5–50 µg/ml). Alexa Fluor 647 fluorophore-conjugated secondary antibodies against the above mentioned species were used at 1∶200 dilution. For membrane staining, transfected cells were detached with trypsin 48 h post-transfection and incubated for 30 min on ice with DyLight 649-conjugated human Fcγ. When needed, cells were subsequently subjected to intracellular staining. A total of 10^4^ cells were analyzed for each histogram using FACSCanto II (Becton Dickinson, Heidelberg, Germany). For detection of membrane exposed RL13, HEK293T were transiently transfected with vectors coding for YFP or RL13-YFP fusion protein, were incubated with different dilutions of mouse monoclonal 5H3/B10 antibody or mouse IgG1 isotype control (Sigma) for 60 min on ice. As secondary antibody, Alexa Fluor 647-conjugated goat anti-mouse was used for 30 min on ice at 1∶200 dilution. Internalization of Fcγ in HEK293T cells was quantified through flow cytometry. 48 h post-transfection cells were detached with trypsin and transferred to round bottom 96 well plates for staining. Cells were washed in cold PBS and incubated on ice with human IgG Fc fragment at different concentrations (50, 25, 12.5 µg/ml). After 30 min incubation, cells were extensively washed and incubated with warm medium to allow for endocytosis (Fcγ_37°C_). Half of the samples were kept on ice for the duration of the experiment (Fcγ_4°C_). At the end of the endocytosis, cells were washed with cold medium and Fcγ remaining on the surface was stained with Alexa fluor 488 conjugated goat anti-human antibody. The percentage of internalized Fcγ was calculated as: (Fcγ_4°C_−Fcγ_37°C_)/Fcγ_4°C_×100. Values shown are relative based on internalization by RL13 wild type.

### Confocal microscopy analysis

Cells transfected with plasmids of interest were trypsin detached and plated on glass coverslips for 24 h post transfection. For intracellular staining, cells were fixed 48 h post transfection with 3.7% paraformaldehyde, permeabilized with 0.1% Triton X-100 (Sigma), blocked with PBS with 1% BSA and stained at RT by incubating for 1 h with primary antibodies in PBS/1% BSA, washed in PBS, and further incubated with secondary antibodies in PBS/1% BSA for 1 h. The ProLong Gold with DAPI (Invitrogen) was used as mounting solution. For membrane staining, the permeabilization step was omitted and fixation was performed after staining with primary and secondary antibodies for 30 min on ice. For human Fcγ internalization, cells expressing RL13-YFP, YFP, or empty vector (controls) were plated on glass coverslips 24 h post transfection. 24 h later, cells were washed in cold PBS and incubated at 4°C with 20 µg/ml of DyLight 649-conjugated human Fcγ for 30 minutes. After extensive washes, temperature was switched to 37°C to allow for internalization. When required, cells were fixed, permeabilized and stained before mounting. The intracellular locations of antibody-tagged or fluorescent fusion proteins were examined under laser illumination in a Zeiss LMS 710 confocal microscope and images were captured using ZEN software (Carl Zeiss).

### Immunoprecipitation

HEK293T cells were transfected with empty vector or myc-His tagged RL13 from TR or Merlin strains. 48 h post-transfection, cells were harvested, washed in cold PBS several times and lysed in lysis buffer (25 mM Tris-HCl pH 7.4, 150 mM NaCl, 1% glycerol, 1 mM EDTA) containing 1% NP-40 (Roche) and supplemented with complete EDTA-free protease inhibitors (Roche). Supernatants were collected after centrifugation at max speed for 30 min at 4°C in a tabletop centrifuge. 100 µg of lysate was incubated with 2 µg of biotinylated human IgG Fc fragment (Jackson immunoresearch) for 1 h at 4°C, then with 30 µl of Streptavidin Dynabeads (Invitrogen) prewashed in lysis buffer. Precipitation was carried out at 4°C with overnight rotation. Immunocomplexes were collected using magnetic beads, washed 4 times with lysis buffer and eluted by adding 30 µl of LDS-buffer and heating at 96°C for 5 minutes. Lysates were mixed with 100 mM DTT (Sigma) and heated at 96°C for 3 min. Proteins were then separated by SDS-PAGE and blotted on a nitrocellulose membrane. Membranes were blocked in blocking buffer (5% w/v nonfat dry milk in PBS with 0.1% Tween 20). All incubations with antibodies were done at room temperature for 1 h in blocking buffer. Mouse anti-His (Invitrogen) was used at 1∶1,000 dilution. Secondary antibodies were diluted 1∶10,000 in blocking buffer.
